# Multifunctional perfluorooctylbromide alginate microcapsules for monitoring of mesenchymal stem cell delivery using CT and MRI

**DOI:** 10.1186/1532-429X-11-S1-O7

**Published:** 2009-01-28

**Authors:** Yingli Fu, Dorota Kedziorek, Ronald Ouwerkerk, Veronica Crisostomo, Wesley Gilson, Nicole Azene, Aravind Arepally, Christine Lorenz, Steven Shea, Robert Krieg, Jeff WM Bulte, Dara L Kraitchman

**Affiliations:** 1 Johns Hopkins University, Baltimore, MD USA; 2 El Centro de Cirugía de Mínima Invasión Jesús Usón, Caceres, Spain; 3 Siemens Corporate Research, Baltimore, MD USA; 4 Siemens Healthcare, Erlangen, Germany

**Keywords:** Mesenchymal Stem Cell, Peripheral Arterial Disease, Human MSCs, Stem Cell Delivery, Peripheral Arterial Disease Patient

## Background and objectives

Many patients with peripheral arterial disease (PAD) cannot undergo conventional medical or surgical therapy due to the extent or severity of atherosclerotic disease. Stem cell therapy has shown promising results as an angiogenic therapy in PAD patients. However, the poor survival of transplanted cells due to early immunodestruction and the inability to noninvasively monitor and track the distribution and proliferation of transplanted cells hinders stem cell therapeutic efficacy. We present here a multifunctional mesenchymal stem cell (MSC) microencapsulation and trafficking method utilizing perfluorooctylbromide (PFOB) incorporated alginate-poly-L-lysine-alginate microcapsules (PFOB Caps) for MSC delivery and noninvasive engraftment tracking using clinical X-ray and MR imaging equipment.

## Methods

Microencapsulation of bone marrow-derived rabbit or human MSCs (1.5 × 10^6^cells/ml) were performed by extruding a PFOB-impregnated 2% (w/v) alginate solution from a syringe pump in conjunction with an electrostatic droplet generator, followed by cross linking with poly-L-lysine to form X-ray- and MRI-visible microcapsules. MSCs viability was examined and compared between unlabeled capsules and PFOB Caps. Using ^19^F MRI and rotational angiograms reconstructed into CT-like images, the minimum detectable concentration was determined in phantoms using standard clinical imaging systems. X-ray delivery and tracking of intramuscular injections of PFOB Caps (~5000 capsules/injection) was assessed in a rabbit PAD model.

## Results

**Figure 1:**
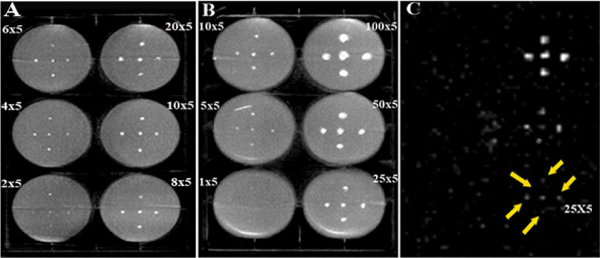
***In vito*** **DynaCT and** ^**19**^**F MR images of PFOB Caps phantoms**. (**A**, **B**) DynaCT images of PFOM Caps phantoms demonstrated the ability to detect as few as 2 capsules. (**C**) ^19^F MRI (3D-TrueFISP, BW = 1500 Hz/px, TR/TE = 3.0/1.5 ms, 2.0 × 2.0 × 5.0 mm^3^, 24 partitions, 4 avgs, 62 s acquisition) of the same phantom as B showed as few as 25 PFOB Caps were identifiable.

The viability of rabbit MSCs encapsulated with PFOB was 90 ± 3% immediately after encapsulation and remained high (88 ± 5% at 4 weeks post-encapsulation). PFOB Caps containing human MSCs had enhanced cell viability relative to unlabeled capsules (83 ± 3% for PFOB vs. 50 ± 1% for control at 65 days post-encapsulation, P < 0.001). Viability of human MSCs in PFOB Caps was maintained up to 100 days, while it decreased sharply to <10% in unlabeled capsules at 80 days post-encapsulation. *In vitro* CT and ^19^F MRI imaging of PFOB Caps demonstrated the ability to detect as few as 2 and 25 capsules (Figure 1), respectively. *In vivo*, PFOB visibility on CT images was demonstrated relative to unlabeled capsules with persistence of intact microcapsules up to 5 weeks post delivery in PAD rabbits.

## Conclusion

By adding PFOB, a dual contrast agent and oxygen carrier, to alginate microcapsules, we have demonstrated the enhanced viability of MSCs within PFOB Caps, and the ability to deliver and track engraftment of stem cells using multiple conventional clinical imaging systems *in vivo*.

